# Factors Influencing the Management of Unruptured Intracranial Aneurysms

**DOI:** 10.7759/cureus.601

**Published:** 2016-05-04

**Authors:** Rebecca L Gillani, Katherine M Podraza, Nijee Luthra, Thomas C Origitano, Michael J Schneck

**Affiliations:** 1 Neurology, Massachusetts General Hospital; 2 Internal Medicine, UC Irvine Medical Center; 3 Neurology, UC Davis Health System; 4 Neurosurgery, Kalispell Regional Healthcare; 5 Neurology, Neurosurgery, Loyola University Chicago, Stritch School of Medicine, Maywood, Illinois

**Keywords:** cerebral aneurysm, decision making, subarachnoid hemorrhage, unruptured intracranial aneurysm

## Abstract

Background

Deciding how to manage an unruptured intracranial aneurysm can be difficult for patients and physicians due to controversies about management. The decision as to when and how to intervene may be variable depending on physicians’ interpretation of available data regarding natural history and morbidity and mortality of interventions. Another significant factor in the decision process is the patients’ conception of the risks of rupture and interventions and the psychological burden of harboring an unruptured intracranial aneurysm.

Objective

To describe which factors are being considered when patients and their physicians decide how to manage unruptured intracranial aneurysms.

Materials & methods

In a retrospective chart review study, we identified patients seen for evaluation of an unruptured intracranial aneurysm. Data was collected regarding patient and aneurysm characteristics. The physician note pertaining to the management decision was reviewed for documented reasons for intervention.

Results

Of 88 patients included, 36 (41%) decided to undergo open or endovascular surgery for at least one unruptured intracranial aneurysm. Multiple aneurysms were present in 14 (16%) patients. Younger patients and current smokers were more likely to undergo surgery, but gender and race did not affect management. Aneurysm size and location strongly influenced management. The most common documented reasons underlying the decision of whether to intervene were the risk of rupture, aneurysm size, and risks of the procedure. For 23 aneurysms (21%), there were no factors documented for the management decision.

Conclusion

The risk of rupture of unruptured intracranial aneurysms may be underestimated by currently available natural history data. Major factors weighed by physicians in management decisions include aneurysm size and location, the patient's age, and medical comorbidities along with the risk of procedural complications. Additional data is needed to define specific aneurysm characteristics and patient factors that influence rupture, in particular in small aneurysms. Physicians should carefully document their rationale along with the patient's perspective given the controversial nature of these management decisions.

## Introduction

Unruptured intracranial aneurysms have a prevalence of approximately three percent and are often discovered as an incidental finding when the patient undergoes magnetic resonance or computed tomography (MRI or CT) imaging for unrelated symptoms [1]. The rationale for treating an unruptured intracranial aneurysm is that rupture may lead to subarachnoid hemorrhage (SAH), often with devastating effects. Approximately 30–40% of patients with SAH will die and a significant portion of survivors (19% in one meta-analysis) will be dependent on activities of daily living (ADLs) [2]. Current options for management of unruptured intracranial aneurysms are observation, or intervention, either by open surgical clipping or endovascular surgery. 

There is still some uncertainty in the medical community about the natural history, i.e. risk of rupture of unruptured intracranial aneurysms. The International Study of Unruptured Intracranial Aneurysm Investigators (ISUIA), published in 2003, found very low rupture rates for aneurysms < 7 mm [3]. However, patients were not randomized to the observation versus treatment arms of the study, causing concerns that the rupture risk may have been underestimated. Critics point to methodological shortcomings of the study and contend that the rupture risk may actually be higher than described in the ISUIA. Given this uncertainty, it can be difficult for patients and physicians to decide how to manage an unruptured intracranial aneurysm.

The decision of how to manage an unruptured intracranial aneurysm involves the careful weighing of the risk of rupture against the risks of treatment, open or endovascular surgery. This includes consideration of patient-specific factors that affect the risk of rupture and comorbidities that affect the risks of treatment. Finally, the personal preferences of the patient must also be considered. While we await more definitive evidence to guide decision-making, these factors are balanced continually by individual physicians and their patients.

With a retrospective chart review study, we aimed to describe some of the factors being considered in the management of unruptured intracranial aneurysms at one academic medical center.

## Materials and methods

Following Loyola Institutional Review Board approval (informed consent waived), we identified all patients who had a hospital or clinic visit at Loyola University Hospital from January 1, 2007–January 1, 2010 and who had ICD9 codes of 747.81 (anomalies of cerebrovascular system), 430 (subarachnoid hemorrhage), or 437.3 (cerebral aneurysm, nonruptured). 517 charts were identified and retrospectively reviewed. To be included, the patient had to have at least one aneurysm managed at Loyola between 9/25/06–01/01/10. Patients were excluded for the following reasons: 216 did not have an aneurysm, 50 presented with subarachnoid hemorrhage (SAH), 48 had a management decision outside of the date limits, 36 had in the medical record only a prior medical history of an intracerebral aneurysm without further recorded details, 33 had no clear management decision, 23 were managed at an outside hospital, eight had fusiform aneurysms, three had recurrent aneurysms, one had a blister aneurysm, and 11 were excluded for multiple of the above reasons. Thus, the total number of cases included in our analysis was 88, and the total number of individual aneurysms analyzed was 111.

Data was collected using standardized forms. Patient characteristics collected included age, sex, race, medical history, use of tobacco, alcohol and illicit drugs, family history, and medications. Data regarding the patients’ aneurysm(s) collected included location, symptoms, date of diagnosis, date of management decision, management, procedure date when applicable, the specialty of managing physician, reason stated in medical record for management decisions, and aneurysm characteristics from MRA, CT angiography, and/or cerebral angiography.

For purposes of data analysis, patients were divided into two groups, procedure (clipping and endovascular surgery) or no procedure. Within the procedure group, aneurysms were treated as follows: 26 clipped, 14 coiled +/- stented, one stented, one coiled and clipped. In cases with more than one aneurysm, the patient was assigned to the procedure group if at least one aneurysm was treated by open or endovascular surgery. Statistical analysis was performed with independent samples t-test, χ2, or analysis of variance as appropriate using SPSS software (IBM Corp., New York, USA) with significance defined at p < .05.

## Results

We examined patient characteristics to determine what characteristics were associated with a decision to undergo open or endovascular surgery. Patients who decided to undergo open or endovascular surgery were significantly younger (t (86) = -4.415, p < .001) and were more likely to currently smoke than patients who chose observation (χ2 (1, N = 88) = 6.364, p =.01) (Table 1).

**Table 1:** Patient Characteristics

Variable	No Procedure (n=52) Number (%)	Procedure (n=36) Number (%)	p
Age	68 ± 14 (SD)	56 ± 11 (SD)	< .001
Gender (female)	39 (75)	30 (83.3)	.35
Race			.31
Caucasian	36 (69.2)	28 (77.8)	
African American	11 (21.2)	4 (11.1)	
Hispanic	3 (5.8)	4 (11.1)	
Current Smoker	9 (17.3)	15 (41.7)	.01
Past Medical History			
Hypertension	40 (76.9)	22 (61.1)	.11
Coronary Artery Disease	12 (23.1)	3 (8.3)	.07
Cardiac Arrhythmias	6 (11.5)	3 (8.3)	.63
Congestive Heart Failure	4 (7.7)	0	.09
Atrial Fibrillation	3 (5.8)	1 (2.8)	.51
Valvular Heart Disease	2 (3.8)	0	.23
Aneurysmal Subarachnoid Hemorrhage	3 (5.8)	5 (13.9)	.19
Family History of Aneurysm	6 (11.5)	8 (22.2)	.18

The majority of patients were women and Caucasian, with no difference between the procedure and no procedure group with regard to gender or race (Table 1). The data was also analyzed to determine the prevalence of cardiovascular diseases as comorbid conditions. Hypertension was common in both groups, with no difference between the groups (Table 1). A small number of patients in both groups had a previous personal history of aneurysmal SAH. There were three in the no procedure group and five in the procedure group. The proportion of patients with a history of an aneurysmal SAH was not significantly different between the two groups. Nor was there a significant difference between the proportions of patients with a family history of aneurysm between the two groups (Table 1).

**Figure 1:**
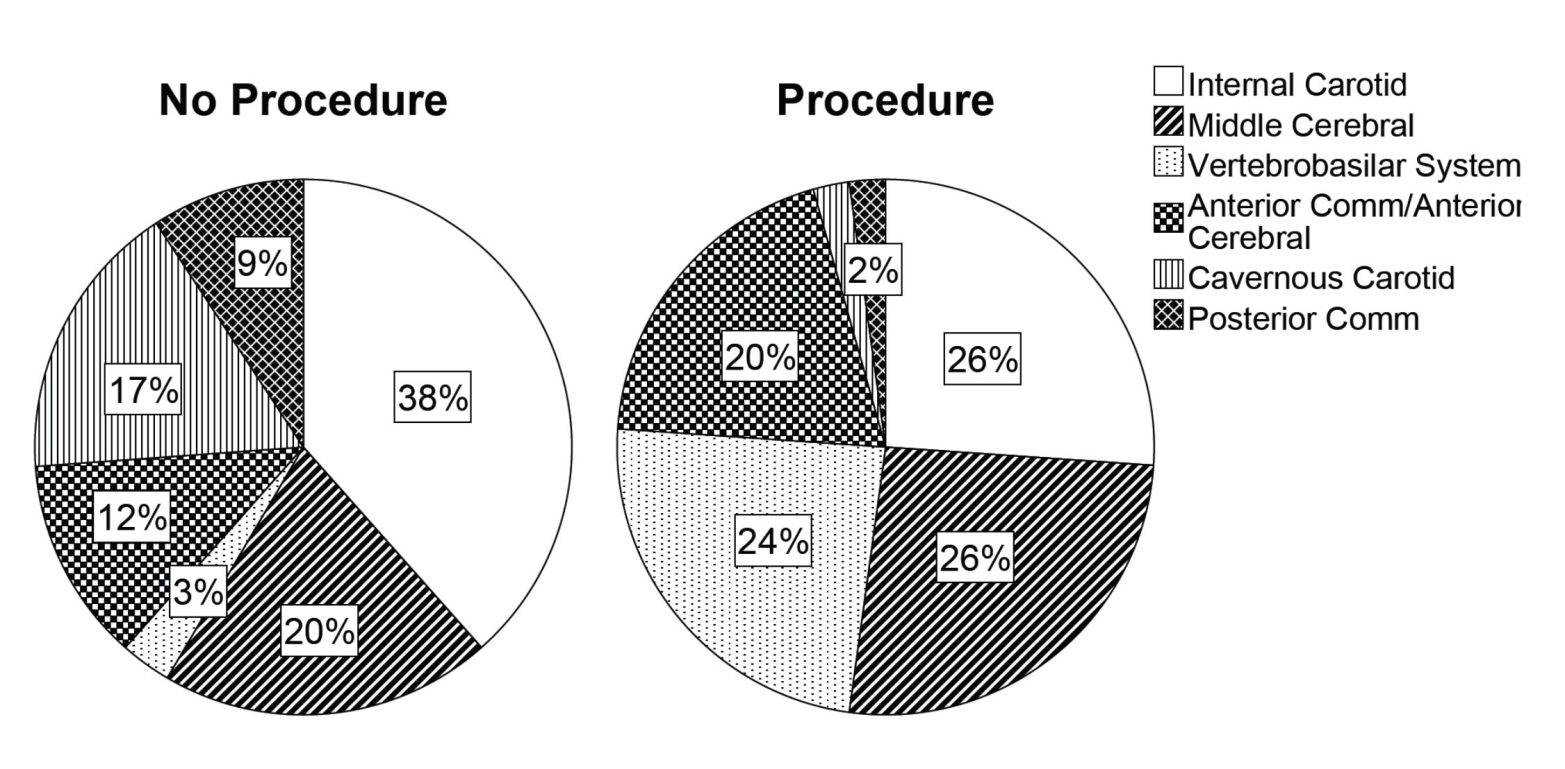
Aneurysm Location by Group Aneurysm location was recorded for all 111 aneurysms in 88 patients. Patients were assigned to the procedure group if the patient decided to undergo open or endovascular surgery, while patients who were observed were assigned to the no procedure group. If a patient had multiple aneurysms, they were assigned to the procedure group if they underwent surgery for at least one of those aneurysms. Pie charts show the distribution of aneurysm locations for patients in the no procedure (n=65 aneurysms), and procedure (n=46 aneurysms) groups. Patients in the procedure group were more likely to have aneurysms in the vertebrobasilar system, while patients in the no procedure group were more likely to have aneurysms of the internal and cavernous carotid arteries (χ2 (5, N = 111) = 20.140, p=.001).

In regard to aneurysm characteristics associated with a decision to undergo open or endovascular surgery, we found no difference in the presence of multiple aneurysms in the no procedure group [7 (13.5%)] or in the procedure group [7 (19.4%)]. In regard to aneurysm location, the location of aneurysms significantly differed between patients who were observed versus those patients who chose to undergo a procedure (χ2 (5, N = 111) = 20.140, p=.001) (Figure 1).

**Figure 2:**
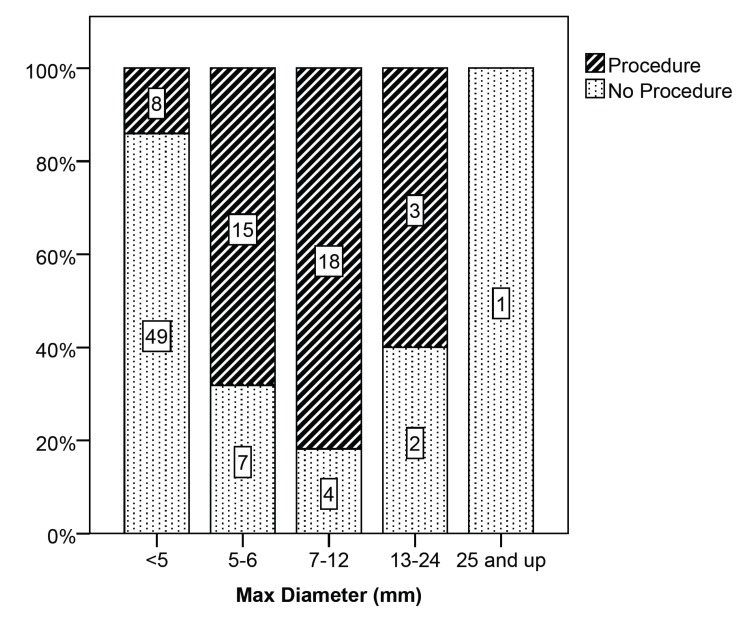
Maximum Aneurysm Diameter by Group Aneurysm size was available in the medical record for 107 (96.4%) aneurysms in 88 patients. Aneurysms were divided by size ranges. For each size range the proportion of aneurysms for patients in the two groups is shown. If a patient had multiple aneurysms, they were assigned to the procedure group if they underwent surgery for at least one of those aneurysms. Number labels on the bars show the number of aneurysms. Small aneurysms <5 mm were more likely to be in patients in the no procedure group. At aneurysm sizes of 5–6 mm and 7–12 mm, patients were more likely to be in the procedure (open or endovascular surgery) group (χ2 (4, N = 107) = 40.410, p< .001).

**Figure 3:**
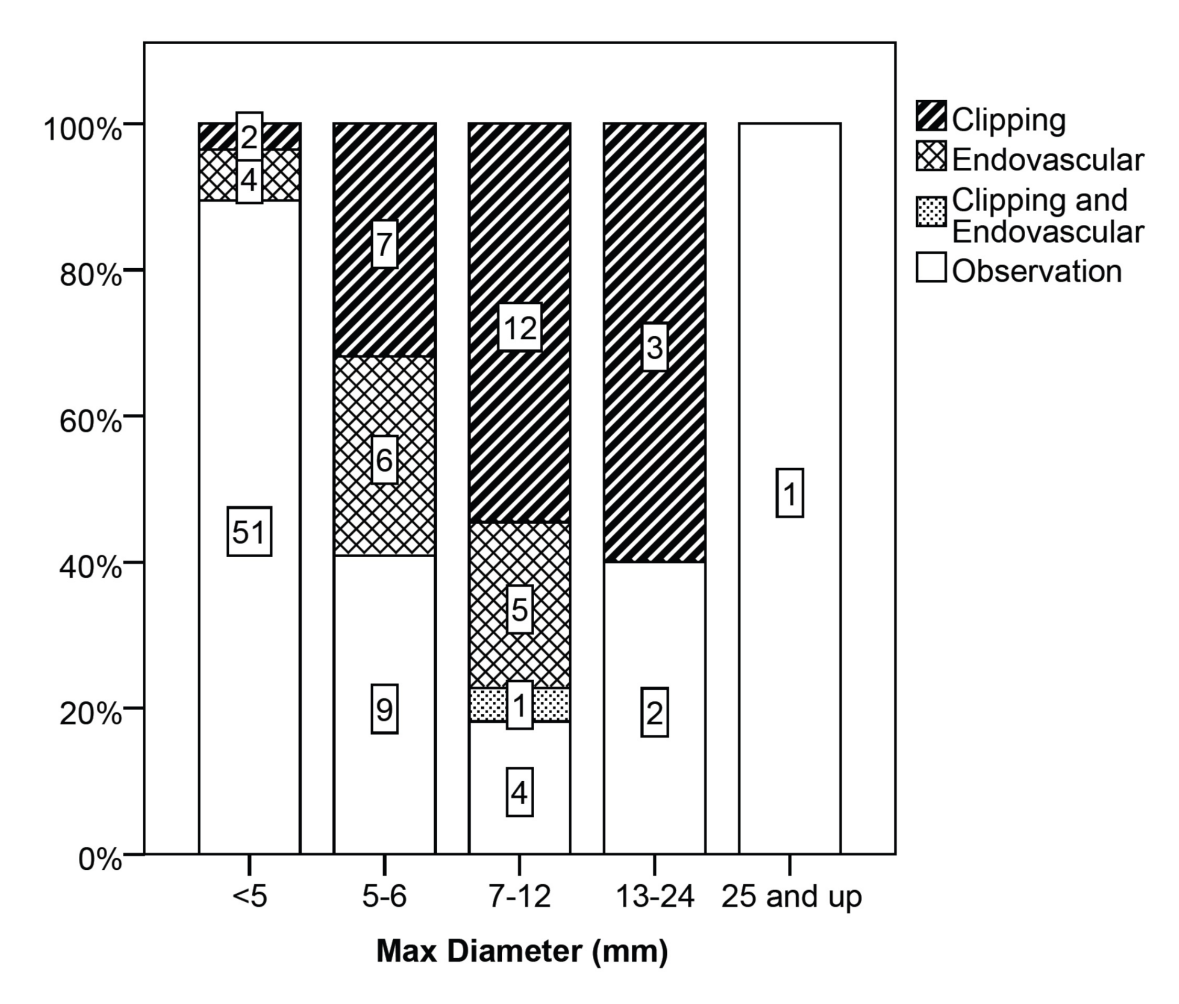
Maximum Aneurysm Diameter by Management Number labels on the bars show the number of aneurysms.

Patients with aneurysms of the internal carotid artery and cavernous carotid artery were more likely to be observed, while patients with aneurysms of the vertebrobasilar system were more likely to undergo a procedure. In regard to maximum aneurysm diameter, patients with larger aneurysms were more likely to undergo open or endovascular surgery (χ2 (4, N = 107) = 40.410, p < .001) (Figure 2-3, which illustrates maximum aneurysm diameter by management).

Slightly more than half of the aneurysms analyzed (57 aneurysms, 53.3%) were <5 mm, and most of these patients (86%) elected not to undergo a procedure. As aneurysm size increased to 5–6 mm, many fewer aneurysms (31.8%) were in patients who were observed, and more aneurysms (68.2%) were in patients who opted for open or endovascular surgery. With increased aneurysm size to 7–12 mm the majority of aneurysms (81.8%) were in patients who chose to undergo surgery. There was a small number of aneurysms with size 13–24 mm and >25 mm, and the three out of six that went untreated were in elderly patients. Two of the untreated aneurysms were in an 86-year-old woman and the third was in a 69-year-old woman with multiple medical comorbidities. When comparing aneurysms that were treated by open as compared to endovascular surgery, there was no difference in maximum aneurysm diameter.

Finally, to better understand the factors that were considered by physicians and patients in the decision of whether to observe or treat aneurysms with open or endovascular surgery, we reviewed clinic notes and hospital notes for documented reasons for the management decision. Common reasons documented were a risk of rupture, aneurysm size, and the risks of a procedure (Table 2).

**Table 2:** Reasons Documented for Management Decision

Factor	Number
Risk of Rupture	45
Size of Aneurysm	36
Risks of Procedure	29
None Stated	23
Comorbid Conditions	16
Patient Wishes	14
Location	13
Benefits of Procedure	13
Consequences of Rupture	11
Age	10
Asymptomatic	10
Risks of Observation	9
Family History	6
Unruptured Aneurysm	6
Projected Longevity	6
Benefits of Observation	5
Symptomatic	3
Not of Clinical Importance	3
Stable Aneurysm	3
Not Source of Symptoms	3
Incidental	2
Smoking Status	2
Aneurysm Morphology	2
Warfarin Use	2
History of Aneurysm Rupture	1
Easily Accessible	1
Neuro Exam Stable	1
Aneurysm Growth	1
Gender	1
Aneurysm Calcification	1
Anti-platelet Use	1

In about a fifth of aneurysms (23 cases, 20.7%) no reason was explicitly stated for the management decision. No reason was explicitly stated for aneurysms in both groups, 17 in the no procedure group and six in the procedure group, and there was no significant difference between groups in the proportion of aneurysms in which no explicit reason for management decision was stated (χ2 (1, N = 111) = 2.818, p = .09). More reasons for the management decision were documented for aneurysms in patients who chose to undergo a procedure (M = 2.89, SD = 1.98) compared to aneurysms in patients who were observed (M = 1.89, SD = 2.001) (t(109) = 2.602, p = .01). With regard to aneurysm size, more reasons for the management decision were documented for aneurysms sized 5–6 mm (3.6) when compared to aneurysms < 5 mm (1.6) (F(3,102) = 7.168, p < .001).

## Discussion

In deciding how to manage an unruptured intracranial aneurysm, patients, and their physicians routinely weigh the risk of rupture against the risks of intervention. This decision is made difficult by the lack of consensus in the medical community on the specific risk of rupture of unruptured intracranial aneurysms. In this study, we describe how 111 unruptured intracranial aneurysms were managed at a single academic center, and reasons documented in the medical record for management decision. In our study, the risk of rupture was the most commonly documented factor in the decision of how to manage an aneurysm. Multiple aneurysm and patient-specific factors are considered by the physicians to attempt to predict the risk of rupture.

The best predictor of the natural history of an unruptured intracranial aneurysm is size. A prospective multi-center study was published by the ISUIA in 2003 and found a low risk of rupture for aneurysms < 7 mm in size (five-year rupture rates of 0% in anterior circulation, 2.5% in posterior circulation and posterior communicating artery) [3]. As aneurysm size increases so does the rupture risk. Other prospective natural history studies have shown slightly higher rupture risk. In a Japanese population, the Unruptured Cerebral Aneurysm Study (UCAS) showed an annual rupture rate for aneurysms < 7 mm in size of 0.23 to 0.31% for the middle cerebral artery and 0 to 0.14% for the internal carotid artery [4]. The highest rates of rupture were in the anterior communicating artery and posterior communicating artery. A smaller study in 142 Finnish patients, most with history of SAH, with an average extended follow-up of 22 years showed an annual rupture rate for aneurysms < 7 mm in size of 0.9% [5].

The shortcoming of these studies is that patients were not randomized to the observation group. It can be argued that there was a selection bias so that patients with low-risk aneurysms were more likely to be observed. Indeed, it has also been shown that the majority of aneurysmal SAH occur in aneurysms < 10 mm in size and very small aneurysms are not infrequently associated with rupture [6]. In our retrospective chart review most patients with very small aneurysms < 5 mm were observed, but the majority of patients who harbored aneurysms > 5 mm underwent treatment. The increased documentation of reasons for the management decision that we observed between the < 5 mm and 5–6 mm groups may reflect the uncertainty in the medical community regarding the risk of rupture of aneurysms in the 5–6 mm group.

Another important predictor of the natural history of an aneurysm is location. Aneurysms in the posterior circulation present a higher risk of rupture [3, 7-8] while those in the cavernous carotid artery are thought to be more ‘benign’ [3]. In our study, most patients with aneurysms of the vertebrobasilar system underwent a procedure, while most patients with cavernous carotid aneurysms were observed and this practice is consistent with the known rupture rates and natural history.

Patient-specific characteristics may influence both the risk of rupture and the morbidity and mortality associated with open or endovascular surgery. Increased rupture risk has been associated with female sex, cigarette smoking, and hypertension [7, 9-10]. We found that smokers and younger patients were more likely to undergo a procedure. The factors that lead to younger patients being more likely to undergo surgery likely include lower morbidity and mortality associated with the open or endovascular surgery, and the longer lifespan that the patient will harbor an aneurysm. By virtue of living more years and harboring the aneurysm for more years, younger patients would potentially have a theoretic higher cumulative risk of rupture.

The patient’s unique perspective will also determine management of unruptured intracranial aneurysms. In our study, the patient’s specific wishes were documented in the medical record as a factor in the management decision in only a minority of cases. However, this likely under-represents the importance of the patient’s perspective in the management decision. For some patients living with a risk of rupture and SAH, a “ticking time bomb,” may be intolerable and lead them to opt for an open or endovascular surgery. A study of the psychosocial effects of living with an unruptured intracranial aneurysm found that those patients with an untreated aneurysm had a small decrease in quality of life as compared to patients with treated aneurysms [11]. The lower quality of life was thought in part secondary to fear about their unruptured intracranial aneurysm. This fear may in part be based on patients overestimating the risk of aneurysm rupture. When neurosurgeons and their patients were surveyed immediately after a consultation, it was discovered that patients overestimated by a factor of two to three the risks of aneurysm natural history and treatment [12].

Overall the documentation in the medical record of reasons underlying the management decision often documented the thought process. However, in 23 cases, no reason was documented. Given that the treatment of unruptured intracranial aneurysms remains controversial, and the consequences of rupture are catastrophic, the physicians caring for these patients should carefully document the specific rationales for management decision.

## Conclusions

There remains uncertainty in the medical community about the natural history, i.e. risk of rupture, of unruptured intracranial aneurysms. There is need for additional data regarding the risk of rupture of small unruptured intracranial aneurysms, and the specific aneurysm characteristics and patient factors that influence rupture. Until more definitive data is obtained, patients and their physicians will continue to weigh the limited available data to make management decisions. In this study, we describe how these management decisions have been made at one academic medical center, where current practice appears to be within the realm of accepted national practice. It remains to be seen how management will evolve with changes in interventional technologies.
